# Investigation of an ALDH1A1-Specific Inhibitor, FSI-TN42, as a Treatment for Obesity in Female Mice

**DOI:** 10.3390/nu18132100

**Published:** 2026-06-27

**Authors:** Jisun Paik, Haley Martin, Andy Jinpyo Kim, Kelsie Neumann, Jessica M. Snyder, John K. Amory

**Affiliations:** 1 Department of Comparative Medicine, University of Washington, Seattle, WA 98195, USA; hmart1n@uw.edu (H.M.); kjp7728@gmail.com (A.J.K.); kelsienn@uw.edu (K.N.); snyderjm@uw.edu (J.M.S.); 2 Department of Medicine, University of Washington, Seattle, WA 98195, USA; amory@uw.edu

**Keywords:** obesity, treatment, ALDH1A1, mouse model

## Abstract

**Background/Objectives**: Retinoic acids (RA) are involved in regulation of weight and energy metabolism. Mice lacking a RA synthesis enzyme, ALDH1A1, are resistant to diet-induced obesity. We previously identified an ALDH1A1-specific inhibitor, FSI-TN42 (N42), and demonstrated its efficacy in suppressing weight gain in male C57BL/6 mice fed a high-fat diet (HFD). In this report, we evaluated whether N42 is similarly effective in female mice. **Methods**: Two studies were performed. In the first study, C57BL/6 female mice were fed a HFD for 12 weeks to induce obesity, after which half were switched to a HFD supplemented with N42 (1 g/kg diet). A control group of mice was maintained on a low-fat purified diet throughout the study. Body weight was determined weekly, and fasting or fed blood glucose was determined at 4–8-week intervals. In the second study, obese female C57BL/6 mice were transitioned from a HFD to either (1) a moderate-fat diet (MFD) or (2) MFD + N42 for 9 weeks. **Results**: N42 significantly suppressed weight gain in female mice maintained on a HFD. However, it did not enhance weight loss when administered alongside a MFD diet, which alone induced significant weight loss comparable to mice fed a control diet throughout the study. **Conclusions**: The ALDH1A1 inhibitor N42 suppresses weight gain in female mice, consistent with prior findings in male mice. However, unlike in males, N42 did not enhance weight loss under conditions of caloric reduction, likely due to more profound weight loss induced by the lower-calorie diet in female mice.

## 1. Introduction

Obesity is a great burden to public health as its comorbidities are chronic diseases requiring lifetime management including diabetes, heart disease, and cancers [1,2,3,4,5]. Long-term weight loss management is challenging, with only lifestyle changes such as diet and exercise for most people. Great progress has been made toward medical options targeting incretin hormones, such as glucagon-like peptide 1 (GLP1) and gastric inhibitory polypeptide (GIP) [6,7]. However, small molecules targeting different pathways can provide alternative options that are cheaper and easier to administer. They may also be used synergistically to improve drug safety and efficacy.

Aldehyde dehydrogenase 1A1 (ALDH1A1, also known as retinal dehydrogenase 1, RALDH1) is an enzyme involved in the second step in a two-step biosynthesis pathway of RA from vitamin A [8]. This is one of the three ALDH1A enzymes responsible for RA synthesis. While ALDH1A2 and 1A3 are indispensable during development [9,10], constitutional loss of ALDH1A1 did not manifest any abnormalities in mice [11]. In addition, *Aldh1a1^−/−^* mice showed resistance to diet-induced obesity [12] mediated partly by increased thermogenesis [13]. While there are controversies regarding the factors and the mechanisms by which ALDH1A1 inhibition suppresses weight gain in mice [12,13,14], both genetic and pharmacological inhibition of ALDH1A1 resulted in decreases in weight gain in mice induced to develop obesity [15,16,17].

We previously demonstrated that an ALDH1A inhibitor, WIN 18,446, can significantly suppress weight gain in male mice fed a HFD [16]. However, WIN 18,446 cannot be used as a treatment for obesity due to non-specific inhibition of all three ALDH1A enzymes as well as ALDH2 that is critical in alcohol metabolism. Broad ALDH1A inhibition by WIN 18,446 results in blockage of spermatogenesis, and its inhibitory effect on ALDH2 leads to a disulfiram reaction upon alcohol consumption. Therefore, we focused our efforts on developing an ALDH1A1-specific inhibitor, as *Aldh1a1^−/−^* mice are fertile and resistant to diet-induced obesity. Previously, we reported the structure and pharmacological characteristics of a novel ALDH1A1-specific inhibitor, N42 [15], and demonstrated that it accelerates weight loss brought on by dietary modification (caloric reduction) in diet-induced obese male mice [17].

The primary goal of current studies was to evaluate the efficacy of N42 on weight loss in female mice to determine if pharmacological ALDH1A inhibition is also efficacious to treat obesity in females. Two studies were conducted to determine if N42 can suppress weight gain in diet-induced obesity and if it can accelerate weight loss when administered with dietary changes to reduce caloric intake. The secondary goal of the studies was to determine if N42 also induce browning of adipose tissue in female mice to confirm a previously demonstrated mechanism by which this drug partly regulates weight in male mice [17].

## 2. Materials and Methods

We conducted two studies to determine the efficacy of N42 as a treatment for obesity in females. No randomization method was used for assigning mice to treatment groups, and the experimenters were not blinded to the treatment groups. The pathologist evaluating histology/immunohistochemistry was blinded to the treatment groups.

### 2.1. Mice and Diets

C57BL/6J female mice were purchased from the Jackson Laboratory (Bar Harbor, ME USA) for both studies and were acclimated in our specific-pathogen-free (SPF) facility [18] for approximately two weeks before study initiation. The mice were housed (*n* = 3/cage) in individually ventilated autoclaved cages with corn cob bedding and nestlets and provided chlorinated reverse osmosis water and maintained with 14:10 (light:dark) cycle. Two studies were performed; the first study was to determine if N42 suppresses weight gain mediated by a high-fat diet, and the second study was to determine if N42 accelerates weight loss induced by a moderate-fat diet. Mice were 7-week-old for the first study and 9-week-old for the second study at study initiation. For the first study, 24 female mice were fed a HFD (TD.220515, 60% calories from fat, Inotiv, Lafayette, IN USA)) for 12 weeks. At 12 weeks, half of these females were switched to a HFD containing N42 (1 g/kg diet), while the other half remained on the HFD for additional 12 weeks. For the second study, 24 female mice were fed the HFD for 12 weeks, and then half of the mice were switched to a moderate fat diet (MFD, TD.220516, Inotiv) while the other half were fed a MFD containing N42 (1 g/kg diet) for an additional 9 weeks. All dietary compositions were reported previously [17]. For both studies, a control group (*n* = 6) was fed a AIN93M, purified diet (TD.00102, Inotiv) for the entire study period.

Mice were weighed weekly and fasting or fed glucose was determined at 4–8-week intervals using OneTouch Glucometer via tail prick. Studies were approved by UW IACUC (protocol number 4038-01).

### 2.2. Histology and Immunohistochemistry

For both studies, all mice were euthanized in the morning by CO_2_ asphyxiation without fasting at the end of the study period. Cardiocentesis was performed, and sera were collected and stored at −80 °C. Liver and adipose depots (perigonadal, retroperitoneal, mesenteric, and inguinal) were dissected and weighed. A small piece of liver and adipose tissues (inguinal fat) was fixed in 10% neutral buffered formalin and processed to paraffin blocks. Tissues were sectioned at 4 microns and stained with hematoxylin and eosin (H&E). UCP1 immunohistochemistry was performed on inguinal adipose tissue sections (Abcam, Waltham, MA, USA, ab23841, 1:1000) as described previously [19]. Adipocyte size was scored by a pathologist blinded to the experimental manipulation; 1, mostly small to medium (<50–75 micron diameter if circular or longest dimension if noncircular); 2, approximately 50% large (approximately 75–100 micron diameter or longest dimension), with multifocal to coalescing regions of smaller adipocytes; 3, at least 75% large; 4, >75% large to very large (>100 micron diameter or longest dimension). Additionally, adipocyte size was quantitated using computer-assisted morphologic analysis. All slides containing inguinal tissue samples were scanned using the Nanozoomer Digital Pathology slide scanner (20X objective, Hamamatsu, Bridgewater, NJ, USA). A tissue detection AI application (Visiopharm, 2025.08.4, Hoersholm, Denmark) was applied to all images to outline tissue sections on each slide (2 sections/slide). A fat membrane detection application was then used to distinguish between cell membrane and background (glass), using a threshold based on hematoxylin values. Post-processing features were used to separate all unique objects (i.e., cells), and cells were labeled based on their size (<400, 400–800, 800–1600, 1600–3200, 3200–6400, and >6400 µm^2^) and counted. The number of cells in each category was normalized against the total cell number and expressed as percent of total cell number. For liver, cytoplasmic vacuolation of the hepatocytes by large or small, clear, and well-delineated vacuoles (consistent with presumptive hepatic lipidosis) was scored according to the following criteria: 0, none; 1, <10% of the liver affected; 2, 10–33% of the liver affected; 3, 34–66% of the liver affected; 4, >66% of the liver affected. Histology and immunohistochemistry were performed through the Histology and Imaging Core at the University of Washington. Images provided in figures were taken using NIS-Elements BR 4.20.01 or BR 5.42.01 64-bit from glass slides and assembled using Adobe Photoshop 2025. Autocorrections were applied to the entire image to adjust white balance, lighting, and/or contrast.

### 2.3. Bulk RNA Sequencing

RNA was extracted from inguinal fats from the first study (30–80 mg) using RNeasy Lipid Tissue Mini Kit (Qiagen, Venlo, The Netherlands) following manufacturer’s instructions. RNA sequencing was performed by Plasmidsaurus (South San Francisco, CA, USA, plasmidsaurus.com) using Illumina sequencing technology and a 3′ end counting approach with custom analysis and annotation. All samples were run as a batch to reduce potential variabilities between runs. RNA-seq analysis was performed by FastQ generation and demux with BCL Convert (v4.3.6) and fqtk (v0.3.1) followed by read-filtering using FastP (v0.24.0) for poly-X tail trimming, 3′ quality-based tail trimming, a minimum Phred quality score of 15, and a minimum length requirement of 50 pb. Alignment to reference genome was performed using STAR aligner (v.2.7) with non-canonical splice junction removal and output of unmapped reads. Gene expression was expressed as gene read counts per million (CPM) generated using featureCounts (subread package v2.1.1) with strand-specific counting, multi-mapping read fractional assignment, exons, and three prime UTR as the feature identifiers and grouped by gene-id and normalized based on sequencing depth.

### 2.4. Statistical Analysis

Statistical analyses were performed using Prism (v10, Graph Pad, La Jolla, CA, USA). Body weight changes were analyzed with repeated-measure two-way ANOVA followed by Tukey’s multiple comparisons between three groups at each time point. Significance was assessed at *p* < 0.05. Liver and fat weight comparisons were carried out with one-way ANOVA followed by Tukey’s multiple comparisons (all groups were compared to each other).

RNA sequencing analysis was performed using proprietary software provided by Plasmidsaurus. DGE analysis was performed using edgePhython (v0.2.5), and correction for multiple testing was performed using the Benjamini–Hochberg methods to control for the false discovery rate (FDR). Significance of DGE was defined by |fold change| ≥ 2 and adjusted *p*-value ≤ 0.05. Functional enrichment was analyzed using the MsigDB Hallmark gene set.

### 2.5. Data Exclusion

In the first study, one mouse enrolled in the HFD group was terminated before reaching the endpoint due to wounds from extreme barbering that could not be treated. Data from this mouse was excluded from analysis. Data from one mouse in the HFD + N42 group was excluded from data analyses as it was identified as a significant outlier throughout the study by the Extreme Studentized Deviate (ESD) method using two-sided Grubbs’ test with alpha of 0.01 (Supplementary Figure S1).

## 3. Results

### 3.1. N42 Suppresses Weight Gain by HFD Consumption in Female Mice

**Figure 1:**
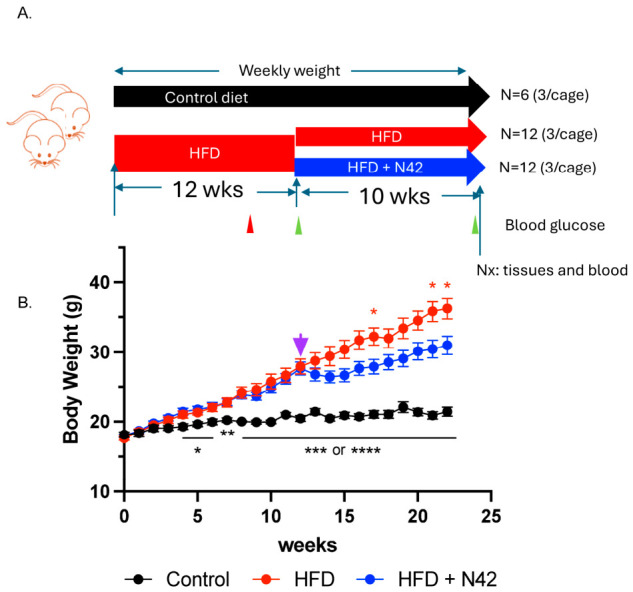
Study design and weekly weight measurement (mean ± SEM). (**A**) Overall study design of the first study. Female mice were fed a HFD for the first 12 weeks. At 12 weeks, half of the HFD group was switched to a HFD containing N42 (blue) while the other half of the mice were continued on HFD alone (red). The control group (black) was fed a low-fat, control diet for the entire study period. Body weight was measured weekly, and blood glucose was determined at week 10 (fasted, red arrowhead), 12, and 22 (fed, green arrowheads). (**B**) Weekly weight measurement (control, *n* = 6; HFD and HFD + N42, *n* = 11/group). Purple arrow indicates start of N42 containing diet. A repeated measure two-way ANOVA test was performed followed by Tukey’s multiple comparisons test at each time point between three groups. Statistical significance was indicated by asterisk (red, HFD throughout the study vs. HFD for the first 12 weeks followed by HFD + N42; black, control vs. HFD or HFD + N42). HFD, high-fat diet. *, *p* < 0.05; **, *p* < 0.01; ***, *p* < 0.001; ****, *p* < 0.0001.

The ALDH1A inhibitors WIN 18,446 and N42 suppressed weight gain in male mice fed a HFD [15,16]. Female mice are known to be resistant to diet-induced obesity compared to males. Thus, we investigated whether a HFD induces obesity and if N42 can suppress weight gain in female mice (Figure 1A). HFD induced significant weight gain in female mice, and weight gain of mice in the HFD group was significantly different from those in the control diet group starting 4 weeks after the diet initiation (Figure 1B). The HFD induced 50% weight gain by 12 weeks and 100% weight gain by 22 weeks. N42 treatment which was initiated at 12 weeks (purple arrow in Figure 1B) significantly suppressed weight gain compared to mice maintained on the HFD. At the end of the study period, N42-treated mice gained 70% weight from the start of the study compared to untreated mice who doubled their weight. Suppression of weight gain in N42 treated mice reached statistical significance at 5 weeks after the N42 treatment compared to mice fed a HFD alone (red asterisk; Repeated measure two-way ANOVA followed by Tukey’s test).

### 3.2. N42 Suppresses Fat Mass Gain and Decreases Hepatic Lipidosis

**Figure 2:**
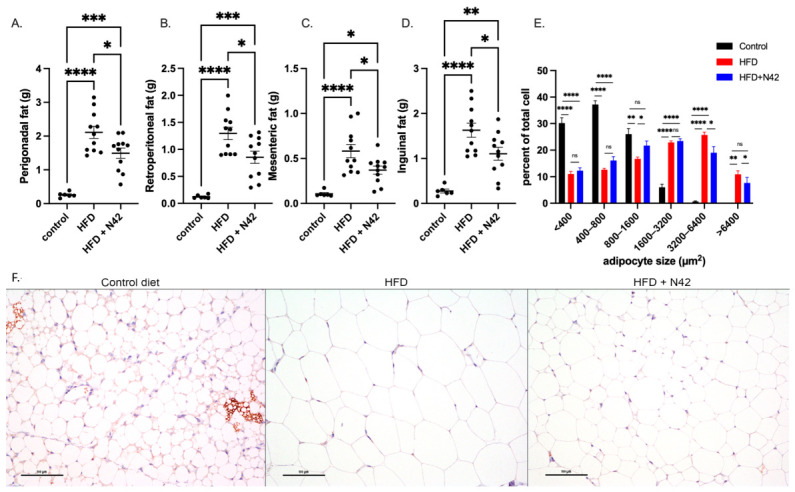
Adipose tissue weight and fat cell size (mean ± SEM). Weights of (**A**) Perigonadal fat; (**B**) Retroperitoneal fat; (**C**) Mesenteric fat; and (**D**) Inguinal fat. (**E**) Inguinal fat cell size distribution. Using computer assisted morphologic analyses, adipocytes were labeled based on their size (<400, 400–800, 800–1600, 1600–3200, 3200–6400, and >6400 µm^2^) and counted. The percentage of each cell size by group is presented. (**F**) Immunohistochemistry of representative inguinal adipose tissue sections (UCP1). Left, control fed diet; Middle, HFD; Right, HFD + N42. Brown indicates anti-UCP1 immunoreactivity; hematoxylin counterstain. Bar is 100 microns. Comparisons among the three groups were made by one-way ANOVA followed by Tukey’s multiple comparisons test. Control, *n* = 6; HFD, *n* = 11; HFD + N42, *n* = 11. *, *p* < 0.05; **, *p* < 0.01; ***, *p* < 0.001; ****, *p* < 0.0001. HFD, high-fat diet.

A HFD causes hepatic lipidosis and fat weight gain. To determine if N42 treatment alters these parameters, we weighed four fat depots at the end of the study period. All four fat depots (perigonadal, retroperitoneal, mesenteric, and inguinal) were significantly increased in mice fed the HFD compared to those fed a control diet, while N42 treatment decreased the weight of all four fat depots compared to HFD alone (Figure 2A–D). Visceral adiposity, calculated by sum of visceral adipose tissue (perigonadal, retroperitoneal, and mesenteric) weight/body weight ×100, was significantly elevated when the mice were fed a HFD compared to control diet but decreased in mice treated with N42 (Supplementary Figure S2A). However, fat depot weights remained higher in mice treated with HFD + N42 compared to the mice fed a control diet. We have previously shown that UCP-1 expression was increased in inguinal fat of mice treated with N42 [17]. Thus, inguinal fat depots were further analyzed using histology (fat cell size) and immunohistochemistry (UCP-1). Fat cell size was significantly increased when mice were fed a HFD compared to mice fed a control diet, while it was significantly decreased in mice fed N42 compared to mice fed a HFD alone (Figure 2E,F, Supplementary Figure S2B,C). Computer-assisted fat cell size analyses showed that N42 treatment shifted fat cell size distribution to the left, i.e., overall smaller sizes (Figure 2E). Semiquantitative scoring by the pathologist agreed well with quantitative scoring (Supplementary Figure S2C), confirming the overall trend. UCP-1 expression was seen in mice fed a control diet but not in mice fed either HFD or HFD with N42 (Figure 2F, Supplementary Figure S2D).

**Figure 3:**
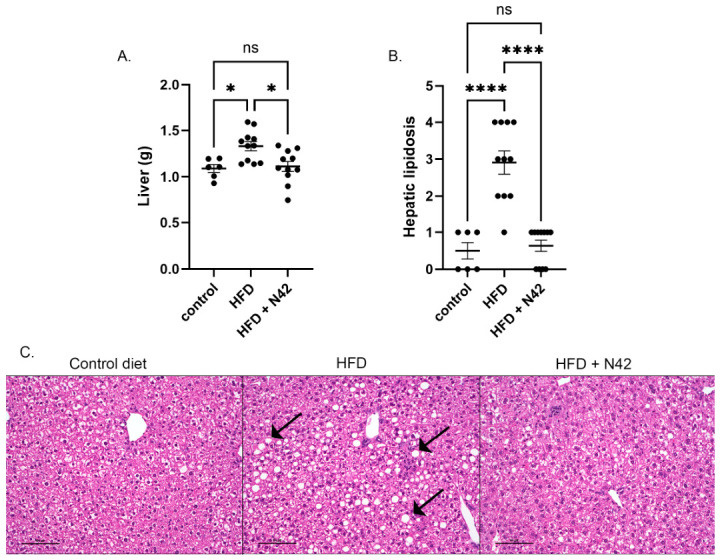
Liver weight and histologic scoring of hepatic lipidosis (mean ± SEM). (**A**) liver weight and (**B**) semi-quantitative scoring of hepatic lipidosis score at the end of the study. (**C**) Histology of liver in control diet fed (**left**); HFD fed (**middle**); and HFD + N42 fed mice (**right**). Arrows indicate clear vacuoles consistent with lipid. Hematoxylin and eosin (HE). Bar is 100 microns. Comparison among the three groups were made by one-way ANOVA followed by Tukey’s multiple comparisons test. Control, *n* = 6; HFD, *n* = 11; HFD +N42, *n* = 11. *, *p* < 0.05; ****, *p* < 0.0001. HFD, high-fat diet.

Liver weight was significantly higher in mice fed a HFD; however, this increase was not observed in mice treated with N42 concomitantly with a HFD, relative to control diet-fed mice (Figure 3A). Hepatic lipidosis scored semi-quantitatively from HE-stained liver sections were also significantly higher in mice fed a HFD but not in mice fed N42 containing HFD compared to mice fed a control diet (Figure 3B,C). All mice in HFD + N42 treated group were scored either 0 (*n* = 4) or 1 (*n* = 11), while mice fed a HFD had a lipidosis score higher than 1, except for one mouse.

### 3.3. N42 Did Not Improve Blood Glucose Levels Despite Decreased Weight Gain When Given with a HFD

**Figure 4:**
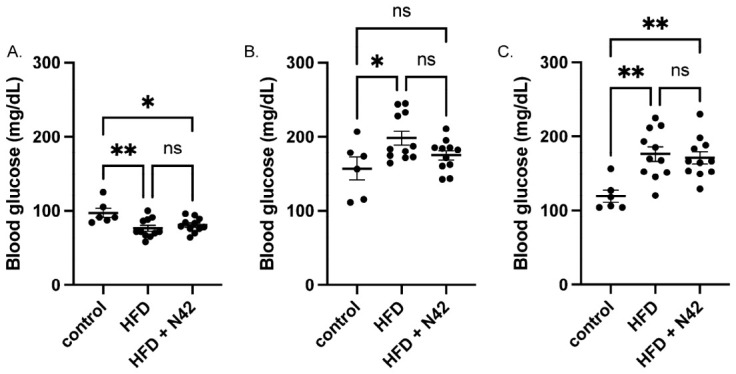
Blood glucose levels (mg/dL). (**A**) fasting blood glucose at week 10. (**B**) Fed blood glucose at week 12. (**C**) Fed blood glucose at week 21. Data are presented as mean ± SEM. One-way ANOVA followed by Tukey’s multiple comparisons were performed. Control, *n* = 6; HFD, *n* = 11, HFD + N42, *n* = 11. *, *p* < 0.05; **, *p* < 0.01, ns, not significant.

Fasting blood glucose levels were normal (<100 mg/dL) at 10 weeks after HFD treatment (Figure 4A), although body weight was significantly higher in mice fed the HFD compared to mice fed a control diet (Figure 1B). We have previously observed that fasting blood glucose levels do not increase significantly, but the fed glucose levels can increase with significant weight changes following a HFD. Thus, we measured blood glucose levels in the morning without overnight fasting. Mice fed a HFD had significantly higher fed blood glucose levels near the end of the treatment period (week 21) compared to mice fed a control diet regardless of N42 treatment (Figure 4C).

### 3.4. N42 Treatment Change Overall Gene Expression Profile in Inguinal Adipose Tissue Compared to HFD Treatment

**Figure 5:**
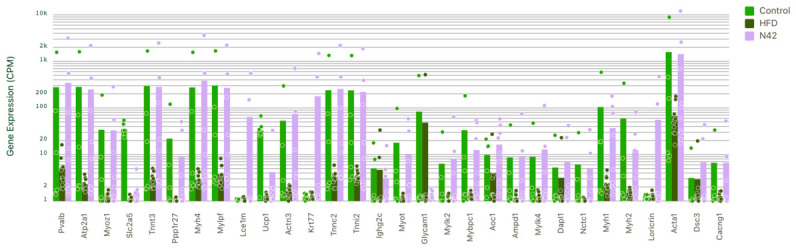
Top 30 most variable genes identified in inguinal adipose tissue across treatment groups. Gene expression levels are presented as count per million (CPM) in log scale.

N42 decreased fat depot weight and fat cell size distribution, although we did not see significant changes in thermogenic gene UCP-1 expression by immunohistochemistry. Thus, we explored global changes in the gene expression profile of the inguinal adipose depots by bulk RNA sequencing. HFD significantly changed the gene expression pattern, resulting in alterations of 1126 genes compared to control diet (Supplementary Figure S3, Supplementary Table S1), of which 586 genes were upregulated with the HFD treatment. N42 treatment with HFD also induced similarly large changes compared to the control diet (Supplementary Figure S4, Supplementary Table S2), resulting in alterations to 816 genes, 401 of which were upregulated with N42 + HFD. When N42 was added to the HFD, only 79 genes were significantly changed compared to HFD (Supplementary Figure S5, Supplementary Table S3), of which 52 genes were upregulated in N42 treated group. The inguinal fat from control-diet-fed mice expressed, at higher levels, genes associated with oxidative phosphorylation, adipogenesis, MTORC1 signaling, and cholesterol homeostasis compared to those from mice fed HFD and HFD + N42. The genes involved in oxidative phosphorylation and adipogenesis were also enriched in the N42 treated group compared to the HFD-treated group. In contrast, inflammatory response or fibroblastic pathway genes are increased in the HFD or HFD + N42 treated groups compared to control. In addition, HFD treated groups had elevated expression of genes involved in the inflammatory response or fibroblastic pathway compared to N42 treated groups. Gene expression patterns show similarities among HFD treated mice, while the mice treated with control or N42 diet were more similar to each other and clustered together (Supplementary Figure S6). Heat map (Supplementary Figure S7) and bar graphs (Figure 5) of 30 top genes that are most variable among the groups also showed that the HFD group is more like each other compared to the control and N42-treated groups. Control and N42-treated mice showed higher expression of genes involved in Ca++ signaling (Cacng1, Atp2a1), muscle contractile (Myh1, Myh2, Myh4, Mylf2), and thermogenesis (Ucp1) (Supplementary Table S4).

### 3.5. N42 Did Not Alter Weight Loss Kinetics or Fat Weight When Provided with a Reduced Calorie, Moderate-Fat Diet

**Figure 6:**
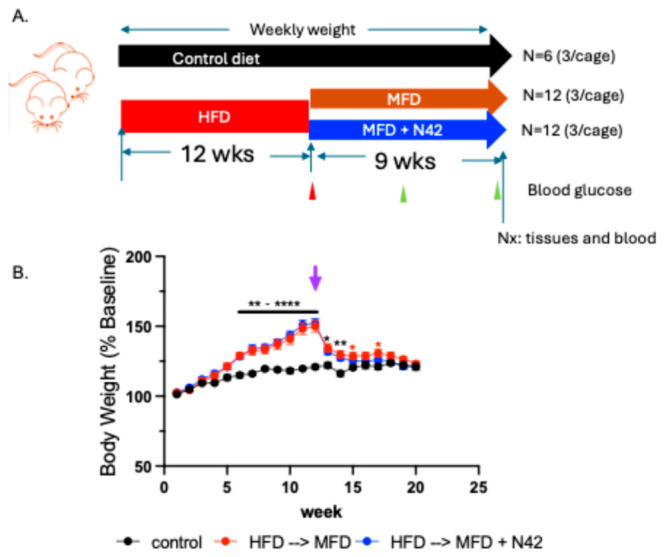
Study design and weekly weight measurement. (**A**) overall study design of the second study. Female mice were fed a HFD for the first 12 weeks. At 12 weeks, half of the mice were switched to a MFD and the other half to a MFD containing N42. The control group was fed a low-fat, control diet for the entire study period. Body weight was measured weekly, and blood glucose was determined at week 12 (fasted, red arrow), 16, and 20 (fed, green arrow). (**B**) Weekly weight measurement expressed as percent of baseline weight (mean ± SEM). Purple arrow indicates the start of MFD (alone) or MFD + N42 diet. A repeated measure two-way ANOVA test was performed followed by Tukey’s multiple comparisons test. *, *p* < 0.05; **, *p* < 0.01; **-****, range of significance between *p* < 0.01–*p* < 0.0001. Black asterisk denotes significance between control vs. HFD –> MFD or HFD –> MFD + N42. Red asterisk denotes comparisons between control vs. HFD –> MFD. HFD, high-fat diet; MFD, moderate-fat diet.

We next tested if N42 can accelerate weight loss when a dietary change (i.e., moderate-fat diet) occurs concomitantly (Figure 6A). HFD induced significant weight gain in female mice, and the MFD induced weight loss regardless of N42 treatment (Figure 6B). By the end of the study period (9 week of MFD), weights of all mice fed the MFD were not significantly different from mice fed a control diet for the entire study period regardless of N42 treatment.

**Figure 7:**
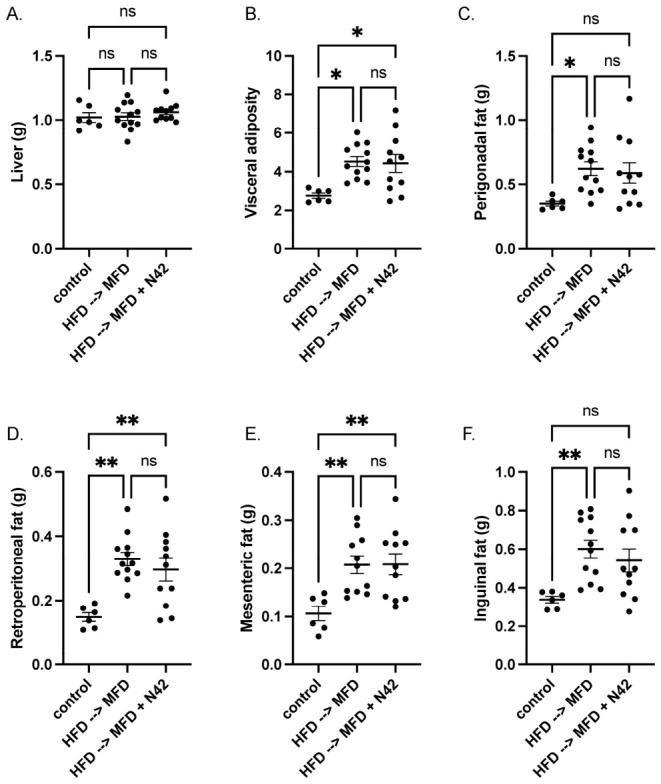
Liver and fat depot weight at the study end period (mean ± SEM). Control, *n* = 6, HFD –> MFD, *n* = 12; HFD –> MFD + N42, *n* = 12 (**A**) Liver weight; (**B**) Visceral adiposity; weight of (**C**) Perigonadal fat; (**D**) Retroperitoneal fat; (**E**) Mesenteric fat; and (**F**) Inguinal fat. Comparisons among the three groups were made by one-way ANOVA followed by Tukey’s multiple comparisons test. *, *p* < 0.05; **, *p* < 0.01.

In accordance with similar weight loss kinetics, N42 did not influence liver weight or adipose tissue weight, resulting in similar liver weight and visceral adiposity between mice fed a MFD vs. MFD + N42 at the end of the study period (Figure 7A,B). Although the percent weight changes were similar among all three groups, adipose tissue weights were still significantly higher in mice initially fed the HFD followed by the MFD compared to those fed the control diet (Figure 7C–F). However, weights of perigonadal and inguinal fat were not significantly different between mice fed the control diet and those fed the HFD followed by MFD + N42 (Figure 7C,F).

### 3.6. N42 Did Not Influence Glucose Metabolism When Provided with MFD

**Figure 8:**
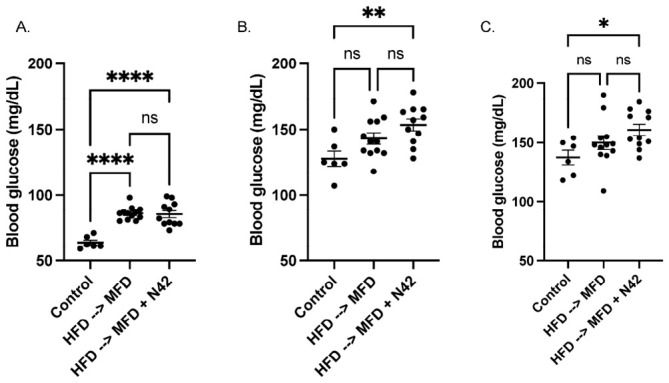
Blood glucose levels (mg/dL). (**A**) Fasting blood glucose at week 12. (**B**) Fed blood glucose at week 16. (**C**) Fed blood glucose at week 20. Data is presented as mean ± SEM. One-way ANOVA followed by Tukey’s multiple comparisons were performed. Control, *n* = 6; MFD, *n* = 12, MFD + N42, *n* = 11. *, *p* < 0.05; **, *p* < 0.01; ****, *p* < 0.0001; ns, not significant.

Fasting blood glucose levels were normal (<100 mg/dL) at 12 weeks after HFD treatment (Figure 8), although they were statistically significantly higher compared to those fed the control diet. Fed glucose levels were elevated at wk 16 and wk 20 (wk 4 and 8 following the MFD or MFD +N42 diet) compared to those from mice fed the control diet. However, N42 treatment did not alter fed blood glucose levels compared to the mice fed the HFD followed by MFD.

## 4. Discussion

The roles of ALDH1A1 in weight regulation have been demonstrated by genetic [12,13,14,20] and pharmacological studies [15,16,17] in both male and female mice, albeit with some conflicting results as well as interpretation.

We have previously reported that pharmacological inhibition of ALDH1A in male mice can suppress weight gain when fed a HFD using the ALDH1A1/1A2 inhibitor WIN 18,446 [16]. We also showed that N42, a specific and potent ALDH1A1 inhibitor, accelerates weight loss when given concurrently with reduced calorie diet, MFD, in a diet- induced model of obesity using male mice [15,17]. It is well known that there are significant sex-dependent differences in weight gain as well as metabolic changes following a HFD in the most-studied mouse strain, C57BL/6 [21,22]. Female mice are generally more resistant to HFD-induced obesity, and thus, they are often excluded in obesity studies. We also previously focused on only male mice in our initial efficacy studies of ALDH1A1 inhibitors to reduce sex-dependent weight and metabolic variances. Our previous studies showed that while WIN 18,446, a pan-inhibitor of ALDH1A1, 1A2, and ALDH2, was very effective in weight suppression, it induced mild hepatic lipidosis and reversibly blocked spermatogenesis [19,23,24]. In contrast, the ALDH1A1 specific inhibitor N42 showed no significant side effects after long-term treatment in males [17]. In current studies, we wanted to test if N42 is efficacious in weight suppression in a diet-induced obesity model in female mice. In agreement with reports in the field, female mice gained weight more slowly when they were fed a HFD compared to males; while male mice double their weight by 8–10 weeks on a HFD [16,17], it took 22 weeks for female mice to double their weight. Similar to what we observed in male mice treated with WIN 18,446 [16], N42 efficiently suppresses weight gain when it is given with a HFD in female mice. Interestingly, N42 treatment significantly reduced hepatic lipidosis (Figure 3B,C), which was not seen in male mice treated with either WIN 18,446 [16] or N42 [17]. Previously, it was reported that female *Aldh1a1^−/−^* mice did not develop hepatic lipidosis when they were fed a HFD compared to wildtype mice [12]. Therefore, it is possible that ALDH1A1 inhibition is more efficacious in preventing lipid accumulation in the liver in females compared to males. However, since N42 was used in male mice only under MFD after obesity induction, lipidosis was not severe in these mice regardless of N42 treatment. Therefore, it is unknown whether the decrease in hepatic lipidosis by N42 treatment under the HFD treatment in females is a sex-specific phenomenon.

We also found differences in effects of ALDH1A1 on adipose tissues between genetic loss of ALDH1A1 (*Aldh1a1^−/−^*) and pharmacological inhibition of ALDH1A1. *Aldh1a1^−/−^* mice showed significant sex-dependent differences in fat weight gain; genetic ALDH1A1 loss induced more significant subcutaneous fat weight loss in males while it induced both subcutaneous and visceral adipose tissue weight loss in females [20]. In contrast, pharmacological inhibition of ALDH1A1 by N42 decreased weight of both visceral and subcutaneous adipose tissue in male [17] and female mice (Figure 2). A possible explanation for these apparent differences may be potential impacts of ALDH1A1 during development in the case of *Aldh1a1^−/−^* mice. Future studies may explore these differences in *Aldh1a1* conditional knock-out mice.

We and others have reported that the loss of ALDH1A1 by genetic [13] or pharmacological means [16,17] induced thermogenic genes in adipose tissues. However, we did not observe increased UCP1 expression by immunohistochemistry in female mice treated with N42 that were housed at room temperature (Figure 2F). UCP-1 expression in the inguinal fat of male mice treated with N42 was more apparent when they were cold challenged compared to those housed in RT [17]. It remains to be determined if cold challenge in female mice would reveal increased UCP-1 expression when the mice are treated with N42.

Browning of white adipose tissues was associated with changes in genes involved in both structure and metabolism [25,26]. The top 30 most differentially expressed genes in inguinal adipose tissue among the three groups were muscle-related genes and calcium signaling-related genes. Because we used whole adipose tissue rather than adipocytes, increased expression of these genes may be due to a higher contribution of stroma-vascular compartments in mice fed a control diet or N42, as their adipocyte size was smaller compared to mice fed a HFD (Figure 2E,F). It can also be interpreted to suggest that adipose tissue from mice fed a control diet or treated with N42 contains more adipocytes with beige potential. Single-cell analysis will be needed to determine these possibilities.

In agreement with previous reports [26], a HFD induces genes with inflammatory and epithelial mesenchymal transition pathway compared to control (Supplementary Figure S3). N42 treatment was associated with relatively lower inflammatory genes and epithelial-mesenchymal-transition-pathway-related genes compared to HFD alone (Supplementary Figure S5). This data suggests to us that while a HFD promotes weight gain and induces inflammatory and fibrotic changes, N42 may counteract some of these effects.

There are several limitations to our studies. The most significant limitation is the lack of detailed functional metabolic assessments to determine the effects of N42 on metabolism in association with weight suppression. While fed or fasting blood glucose levels did not change after N42 treatment, it is possible that more rigorous analyses of glucose tolerance and/or insulin sensitivity could reveal the impact of N42 on metabolism. We also did not analyze food intake or overall metabolic changes using indirect calorimetry, and thus it is not possible to show the mechanisms by which weight suppression occurred. We did not perform a prior power calculation which required mean and variance of weight in females under a HFD. Instead, we estimated the animal number needed to see significant weight changes in response to HFD based on the literature [20]. The chosen animal number (*n* = 12/group) was sufficient to show weight changes following N42 treatment but may not have been sufficient to determine more subtle changes in metabolic parameters such as blood glucose levels or in gene expression. Finally, while we attempted to evenly distribute the weight of the mice between the two treatment groups matching the group average weight following a HFD before N42 treatment began, complete randomization or blinding were not possible as diets were distinguished by different coloring agents (HFD vs. HFD + N42 or MFD vs MFD + N42). However, most of the outcome measures were objective, i.e., body weight or organ weight, limiting bias that could occur with subjective evaluation. We tried to reduce bias in subjective evaluation such as histological scoring by blinding the pathologist to the treatment groups.

## 5. Conclusions

In summary, our studies demonstrate that an ALDH1A1 inhibitor, N42, is also efficacious in suppressing weight gain in female mice and brings about gene expression changes associated with decreased inflammation and increased oxidative phosphorylation. However, we could not determine if N42 treatment can accelerate weight loss brought on by dietary changes (MFD) in female mice because female mice lost weight rapidly upon this dietary change, reaching levels similar to mice fed a control diet. We also found that N42 can suppress hepatic lipidosis in HFD treated animals. We do not know if this is a secondary effect to suppression of weight gain or independent of weight suppression. Future studies may include investigation into its efficacy on metabolic-dysfunction-associated steatohepatitis (MASH). Further studies will also be needed for determining mechanisms by which N42 induce changes in adipose tissue.

## Data Availability

All data generated or analyzed during this study are included in this published article and its Supplementary Information files.

## Supplementary Materials

The following supporting information can be downloaded at: https://www.mdpi.com/article/10.3390/nu18132100/s1, Figure S1: Weight distribution of mice assigned to each diet group following a HFD at weeks between 5 and 12; Figure S2: Visceral adiposity and inguinal adipose tissue analyses; Figure S3: Gene expression in inguinal fat of control vs. HFD fed mice; Figure S4: Gene expression in inguinal fat of control vs. HFD + N42 fed mice; Figure S5: Gene expression in inguinal fat of HFD vs. HFD +N42 fed mice; Figure S6: Heat map of overall gene expression profiles of inguinal fat; Figure S7: Heat map of 30 most variable genes in inguinal fat; Table S1: Differentially expressed genes, control vs. HFD; Table S2: Differentially expressed genes, control vs. HFD + N42; Table S3: Differentially expressed genes, HFD vs. HFD + N42; Table S4: Top 30 variable genes.

## Abbreviations

The following abbreviations are used in this manuscript:

RA	Retinoic acid
HFD	High fat diet
MFD	Moderate fat diet
ALDH1A1	Aldehyde dehydrogenase 1A1
H&E	Hematoxylin & Eosin
CPM	Count per million
UCP1	Uncoupling protein 1
