# Supplementary material for: Investigation of an ALDH1A1-Specific Inhibitor, FSI-TN42, as a Treatment for Obesity in Female Mice
Source: Nutrients. 2026 Jun 27;18(13):2100. doi: 10.3390/nu18132100 (PMC13362916; doi:10.3390/nu18132100)
Supplement: Supplementary file 1 [file nutrients-18-02100-s001.zip › Supplementary figures.pptx]

## Slide 1
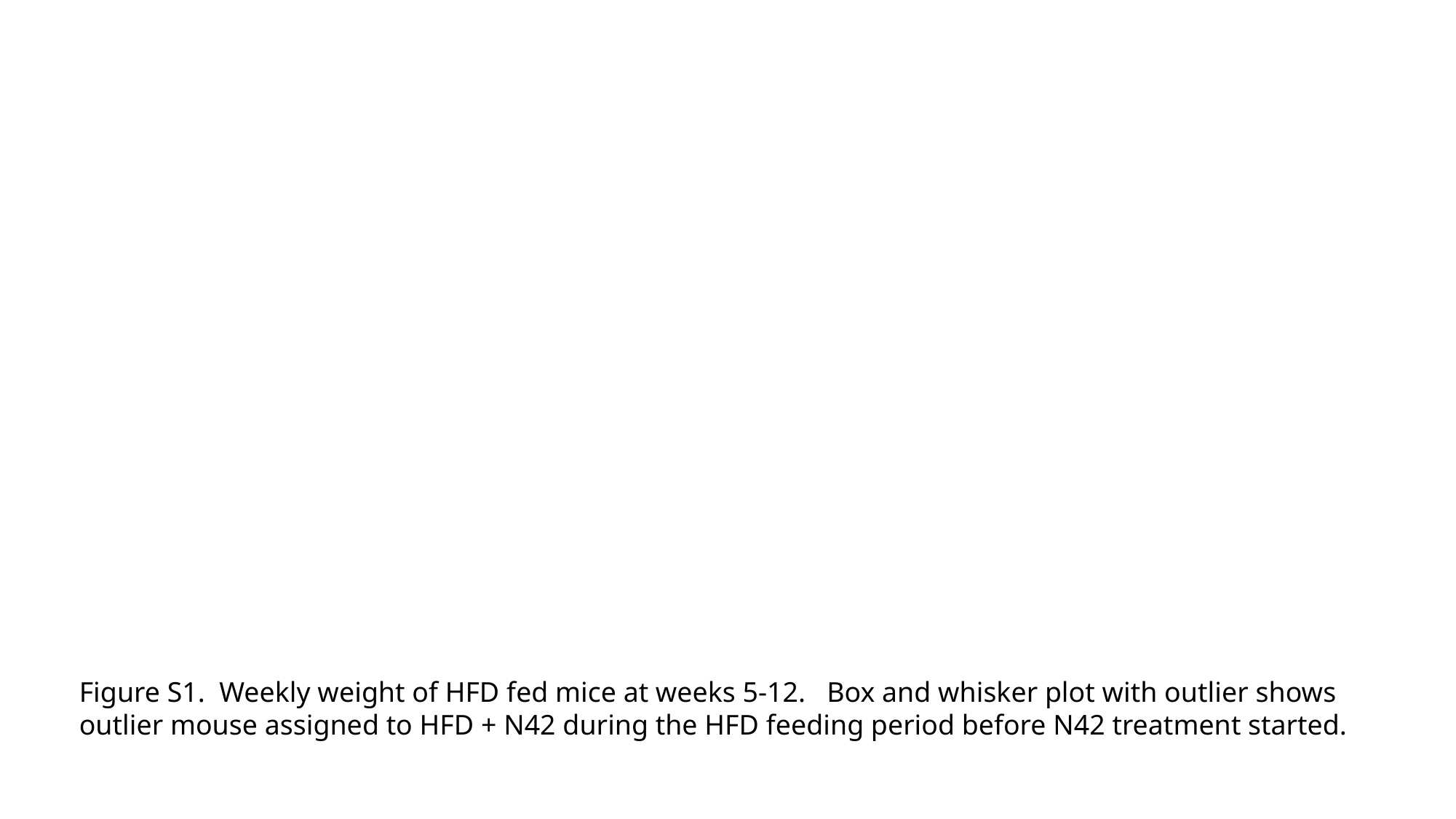

Figure S1. Weekly weight of HFD fed mice at weeks 5-12. Box and whisker plot with outlier shows outlier mouse assigned to HFD + N42 during the HFD feeding period before N42 treatment started.

## Slide 2
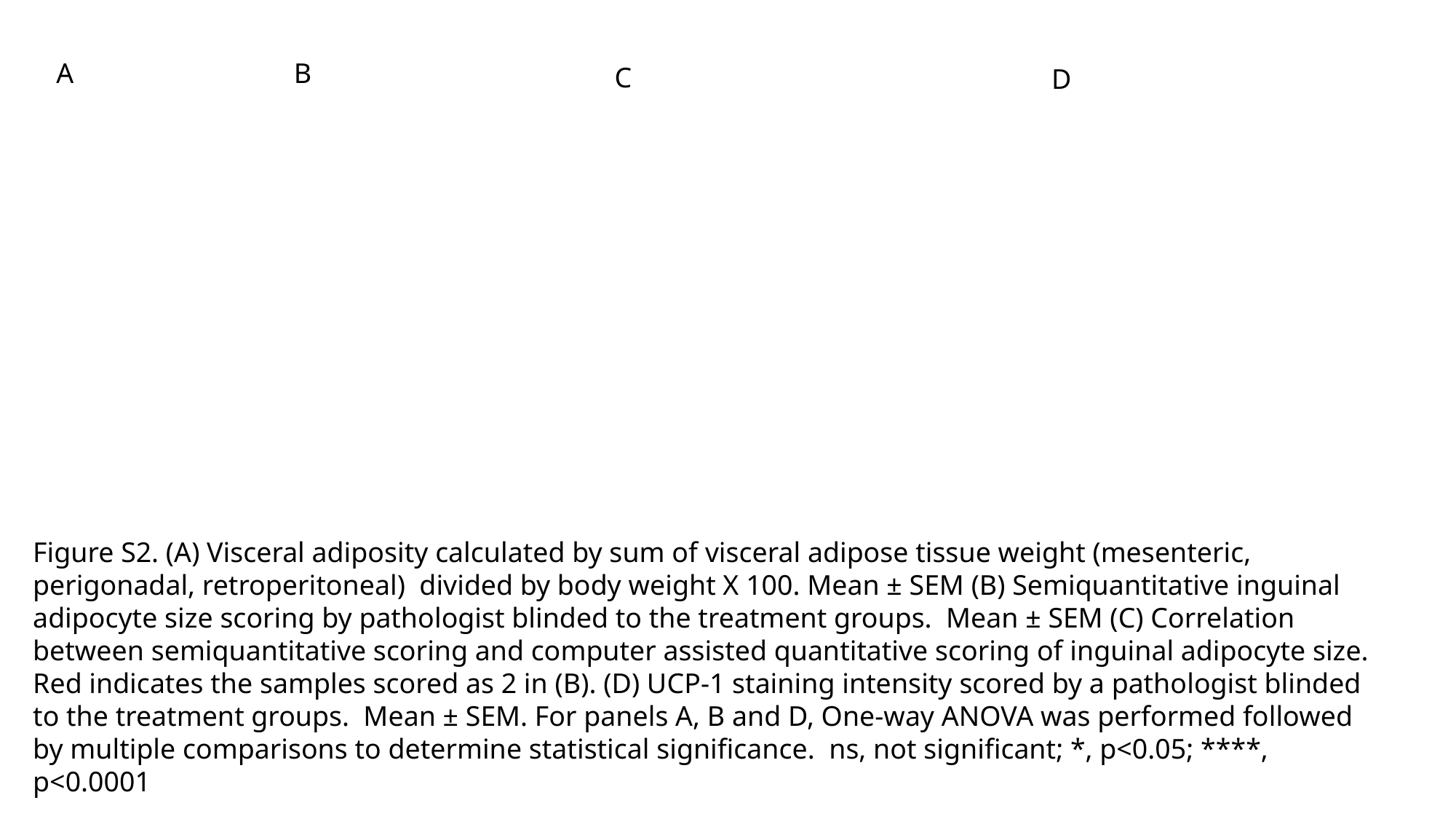

A
B
C
D
Figure S2. (A) Visceral adiposity calculated by sum of visceral adipose tissue weight (mesenteric, perigonadal, retroperitoneal) divided by body weight X 100. Mean ± SEM (B) Semiquantitative inguinal adipocyte size scoring by pathologist blinded to the treatment groups. Mean ± SEM (C) Correlation between semiquantitative scoring and computer assisted quantitative scoring of inguinal adipocyte size. Red indicates the samples scored as 2 in (B). (D) UCP-1 staining intensity scored by a pathologist blinded to the treatment groups. Mean ± SEM. For panels A, B and D, One-way ANOVA was performed followed by multiple comparisons to determine statistical significance. ns, not significant; *, p<0.05; ****, p<0.0001

## Slide 3
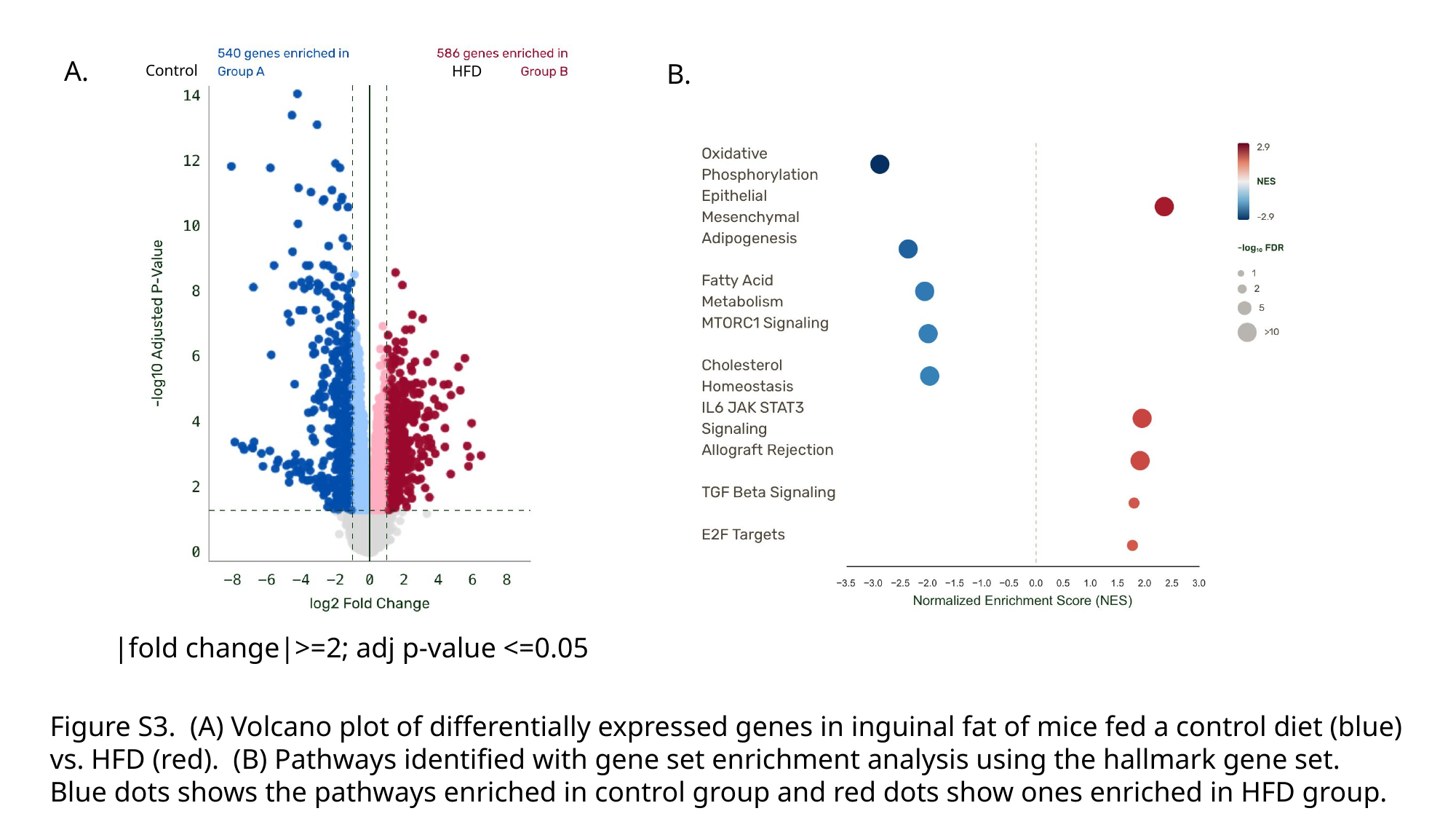

A.
B.
Control
HFD
|fold change|>=2; adj p-value <=0.05
Figure S3. (A) Volcano plot of differentially expressed genes in inguinal fat of mice fed a control diet (blue) vs. HFD (red). (B) Pathways identified with gene set enrichment analysis using the hallmark gene set. Blue dots shows the pathways enriched in control group and red dots show ones enriched in HFD group.

## Slide 4
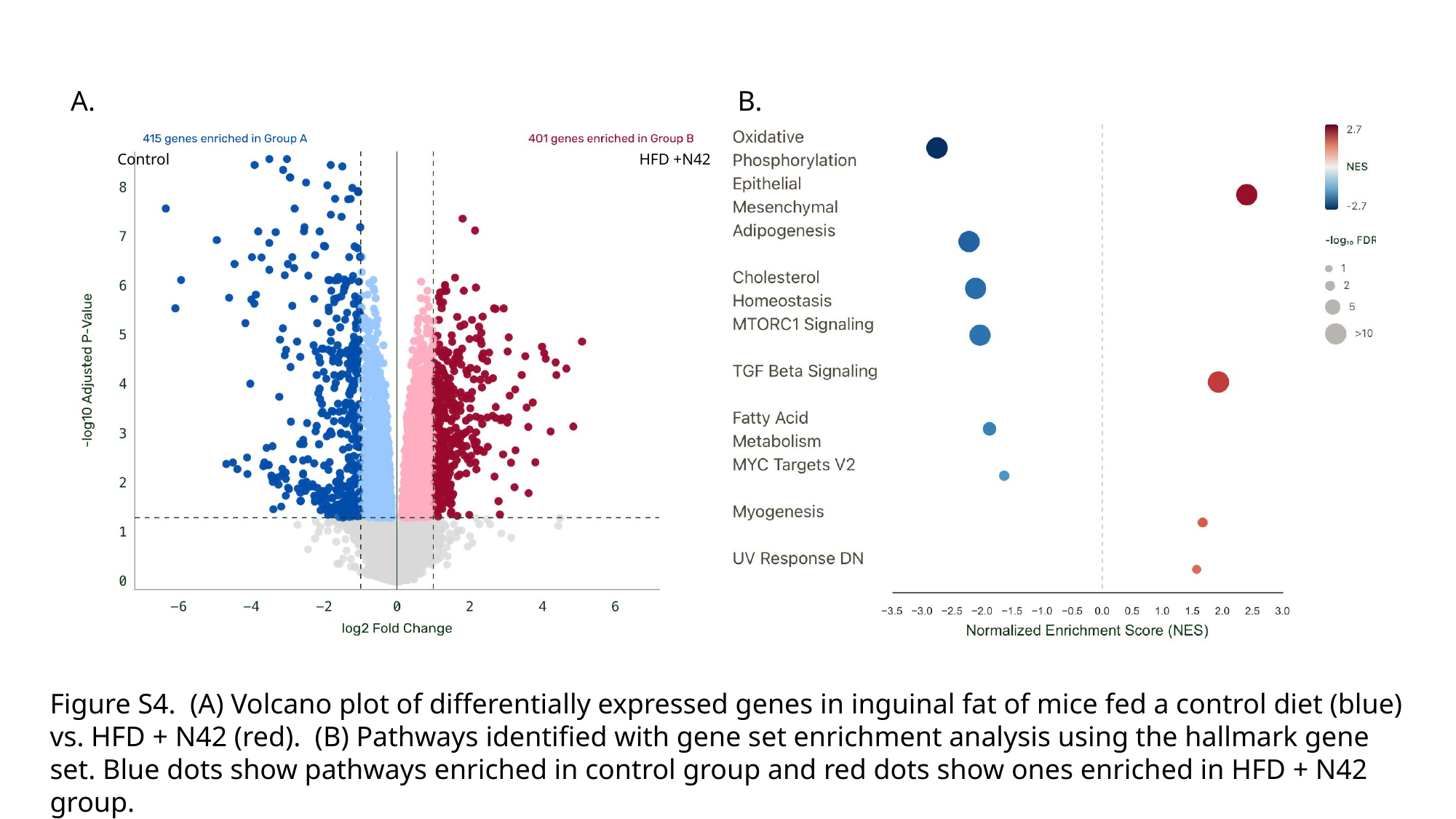

A.
B.
Control
HFD +N42
Figure S4. (A) Volcano plot of differentially expressed genes in inguinal fat of mice fed a control diet (blue) vs. HFD + N42 (red). (B) Pathways identified with gene set enrichment analysis using the hallmark gene set. Blue dots show pathways enriched in control group and red dots show ones enriched in HFD + N42 group.

## Slide 5
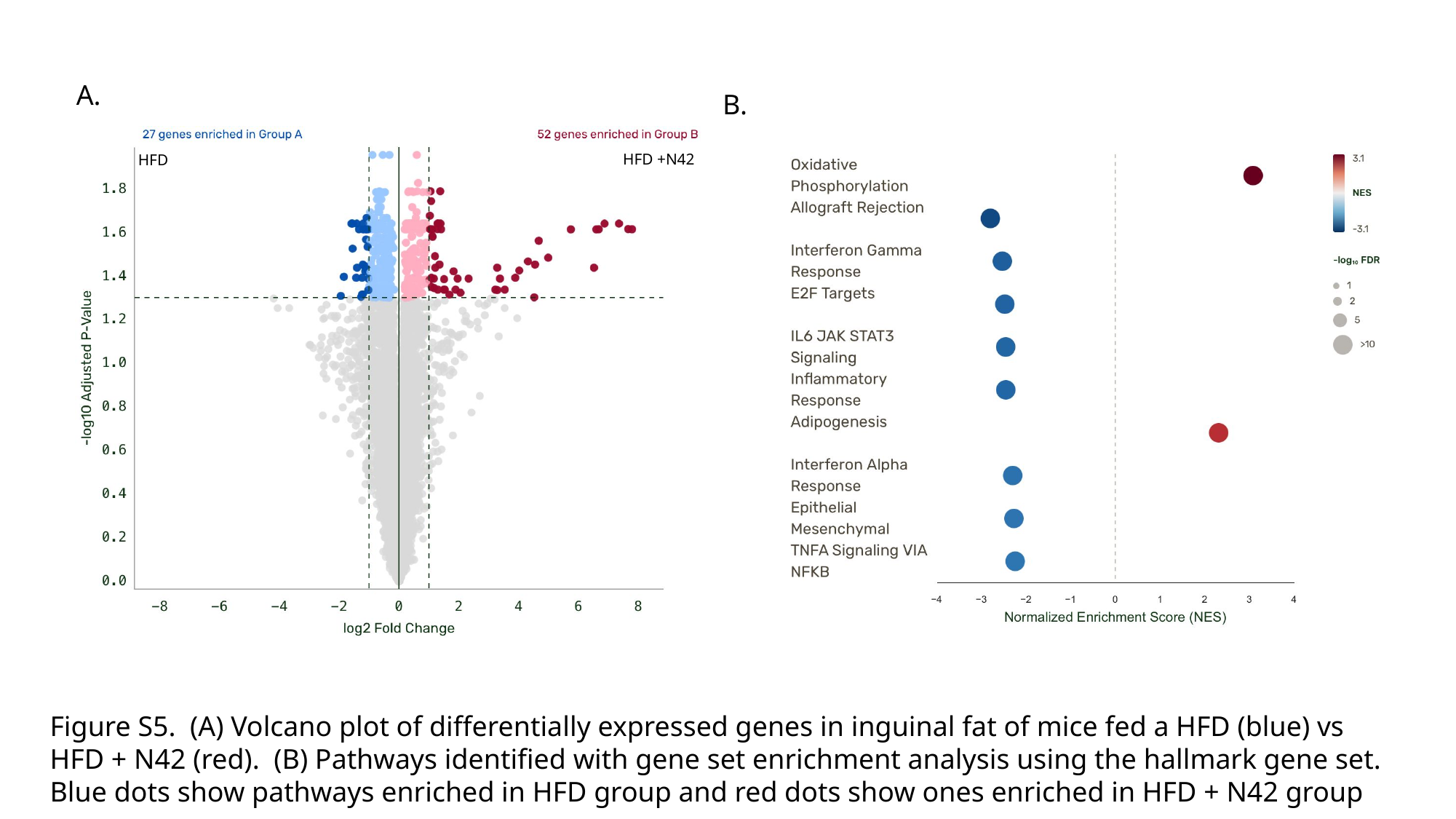

A.
B.
HFD +N42
HFD
Figure S5. (A) Volcano plot of differentially expressed genes in inguinal fat of mice fed a HFD (blue) vs HFD + N42 (red). (B) Pathways identified with gene set enrichment analysis using the hallmark gene set. Blue dots show pathways enriched in HFD group and red dots show ones enriched in HFD + N42 group

## Slide 6
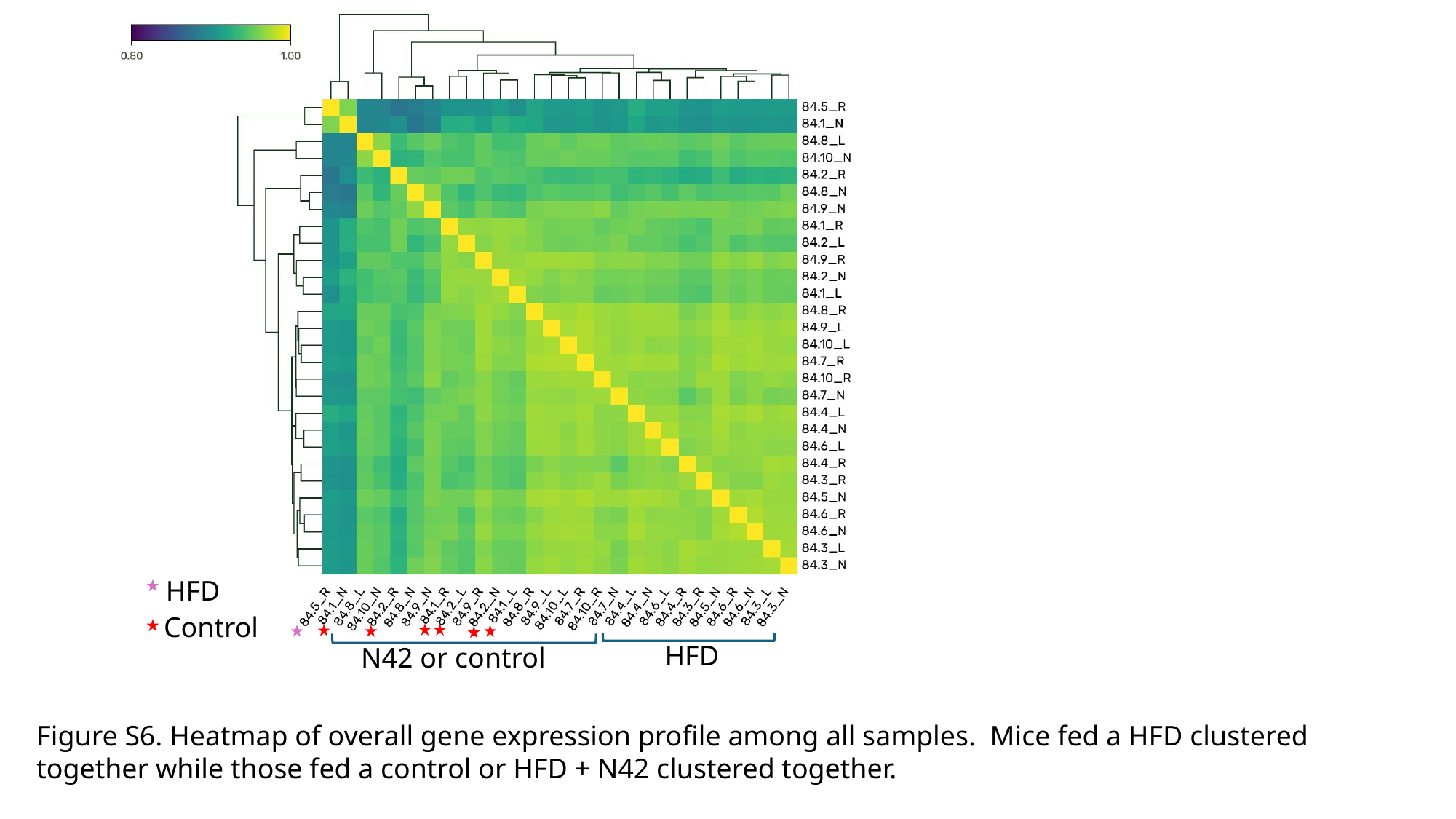

HFD
Control
HFD
N42 or control
Figure S6. Heatmap of overall gene expression profile among all samples. Mice fed a HFD clustered together while those fed a control or HFD + N42 clustered together.

## Slide 7
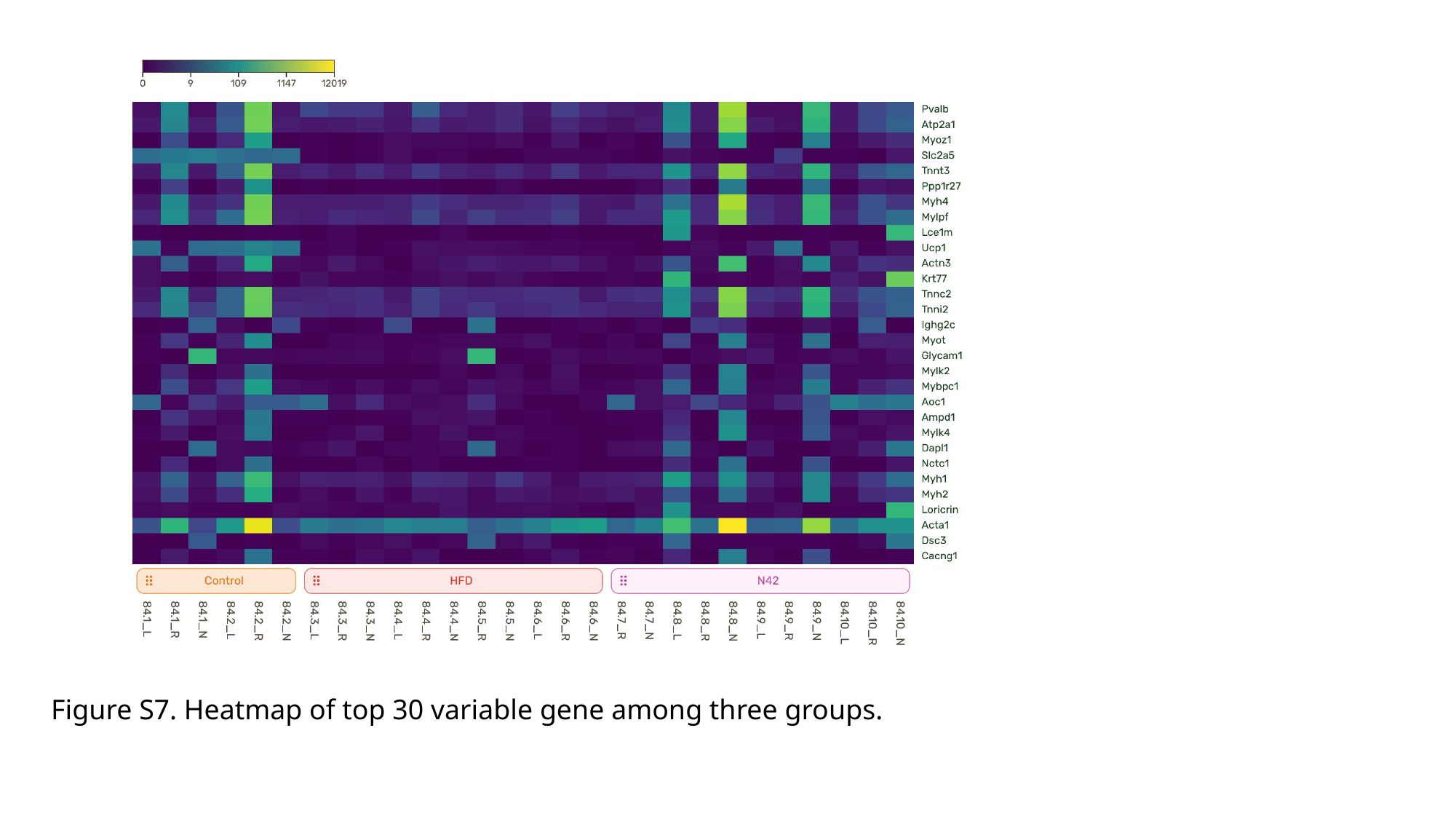

Figure S7. Heatmap of top 30 variable gene among three groups.
